# Comparative Analysis of Diagnostic Efficacy in Primary Hyperparathyroidism: A Comparison Analysis of ^11^C-Choline PET/CT, Neck Ultrasonography, ^99m^Tc-MIBI Dual-Phase Planar Scintigraphy, and ^99m^Tc-MIBI SPECT/CT Imaging

**DOI:** 10.7150/jca.91483

**Published:** 2024-02-04

**Authors:** ZhiNing Zhang, Min Zhao, Bin Chen, YiMing Cao, Shi Gao

**Affiliations:** Department of Nuclear Medicine, China-Japan Union Hospital of Jilin University, Changchun 130033, China.

**Keywords:** primary hyperparathyroidism, ^11^C-choline, ^99m^Tc-MIBI, neck ultrasonography, SPECT, PET

## Abstract

**Objective:** To compare the diagnostic efficacy of ^11^C-choline PET/CT, neck ultrasonography, ^99m^Tc-MIBI dual-phase planar scintigraphy, and ^99m^Tc-MIBI SPECT/CT imaging in the diagnosis of primary hyperparathyroidism (PHPT).

**Methods:** We conducted a retrospective analysis of 32 patients with PHPT who visited the Nuclear Medicine Department of Jilin University China-Japan Union Hospital between January 2019 and December 2022. All patients underwent ^11^C-choline PET/CT, neck ultrasonography, ^99m^Tc-MIBI dual-phase planar scintigraphy, and ^99m^Tc-MIBI SPECT/CT examinations within two months before surgery. Sensitivity, specificity, positive predictive value, and negative predictive value of each imaging study were compared using postoperative pathology and follow-up results. Diagnostic efficacy was further analyzed using ROC curve analysis. Factors influencing on ^99m^Tc-MIBI imaging were also investigated.

**Results:** A total of 35 lesions were resected in the 32 patients. The diagnostic sensitivity of ^11^C-choline PET/CT, neck ultrasonography, ^99m^Tc-MIBI dual-phase planar scintigraphy, and ^99m^Tc-MIBI SPECT/CT was 88.2%, 52.9%, 58.8%, and 67.6%, respectively. Specificity was 96.8%, 95.7%, 96.8%, and 95.7%, respectively. Positive predictive values were 90.9%, 81.8%, 86.9%, and 85.2%, respectively, and negative predictive values were 95.7%, 84.9%, 86.7%, and 89.1%, respectively. The areas under the ROC curve (AUC) were 0.925, 0.743, 0.778, and 0.817, respectively. Among them, ^11^C-choline PET/CT had higher sensitivity and AUC than other imaging studies (p<0.05), while specificity, positive predictive value, and negative predictive value were similar (p>0.05). The positive group in ^99m^Tc-MIBI SPECT/CT imaging had significantly larger lesion diameters than the negative group (P<0.05), while preoperative blood calcium and PTH showed no statistical differences (P>0.05).

**Conclusion:** ^11^C-choline PET/CT demonstrates superior preoperative diagnostic efficacy for PHPT compared to neck ultrasonography, ^99m^Tc-MIBI dual-phase planar scintigraphy, and ^99m^Tc-MIBI SPECT/CT. Lesion size may be the primary factor affecting the sensitivity of ^99m^Tc-MIBI imaging.

## Introduction

Primary hyperparathyroidism (PHPT) is the third most common endocrine disorder after diabetes and hyperthyroidism. Epidemiological data indicate an incidence of approximately 1/1000 to 1/500, with females being affected 2-3 times more than males 1, 2. PHPT typically manifests as a clinical syndrome characterized by muscle weakness, joint pain, kidney stones, and osteoporosis. In severe cases, it can lead to brown tumors, pathological fractures, recurrent gastrointestinal ulcers, and neurological symptoms, significantly affecting the physical and mental health as well as the quality of life of affected individuals. Laboratory tests reveal elevated blood levels of parathyroid hormone (PTH), increased calcium levels, and decreased phosphorus levels in patients with this condition. However, PHPT often presents with subtle symptoms and a wide range of clinical manifestations. Especially in recent years, with the increasing popularity of routine health check-ups, the clinical manifestations of PHPT gradually shift towards mild or even asymptomatic symptoms, making it more challenging to establish a definitive diagnosis 3.

Currently, surgery is the only effective means of curing PHPT, while drug therapy is reserved for a minority of patients who cannot tolerate or refuse surgery. In recent years, the increasing popularity of minimally invasive parathyroidectomy (MIP) has replaced traditional bilateral neck exploration (BNE) as the mainstream approach for treating PHPT, thanks to the widespread adoption of techniques like laparoscopy and robotics 4. Although this newer approach is associated with fewer traumas and a lower complication rate, it presents a new challenge due to limitations in the surgical field of view, requiring a higher degree of precision from the surgeon. Therefore, precise preoperative localization is essential for patients undergoing MIP surgery.

Various imaging techniques have been applied clinically for lesion diagnosis and localization in PHPT patients. These include morphology-based methods such as ultrasound, CT, and MRI, as well as functional approaches like nuclear medicine SPECT and PET imaging. Among these, neck ultrasound and ^99m^Tc-MIBI parathyroid imaging, as classical imaging methods, hold significant clinical value and are still recommended as primary preoperative localization techniques in PHPT by various international guidelines. Initially employed for prostate cancer diagnosis with favorable diagnostic efficacy, ^11^C-choline PET/CT imaging was incidentally discovered to be applicable for PHPT localization in 2014. It has garnered increasing attention in recent years 5. However, comprehensive comparative studies assessing the diagnostic efficacy of ^11^C-choline PET/CT imaging versus traditional imaging methods in PHPT are currently lacking. In this study, we aim to thoroughly explore the diagnostic value of ^11^C-choline PET/CT for preoperative localization in PHPT by conducting comparative analyses with neck ultrasound, ^99m^Tc-MIBI dual-phase planar imaging, and ^99m^Tc-MIBI SPECT/CT. Additi onally, we investigate factors that may influence the results of ^99m^Tc-MIBI imaging.

## Patients and methods

### Patient population and selection

A retrospective analysis was conducted on PHPT patients who visited Jilin University China-Japan Union Hospital from January 2019 to December 2022. All patients underwent preoperative imaging studies, including neck ultrasound, ^99m^Tc-MIBI dual-phase planar imaging, ^99m^Tc-MIBI SPECT/CT, and ^11^C-choline PET/CT, all of which were completed within 2 weeks. Prior to the examinations, none of the patients had received surgical or endocrine treatment. Within 2 months after the examinations, the patients underwent surgical treatment in our hospital's thyroid surgery department. Inclusion criteria: 1) Patients were diagnosed with PHPT based on comprehensive analysis by clinical physicians from the thyroid surgery and endocrinology departments, as well as biochemical tests including PTH, calcium, and phosphorus levels. 2) Patients were assessed by thyroid surgery physicians who confirmed the need for surgery and the ability to tolerate it. Exclusion criteria: 1) Incomplete preoperative imaging studies. 2) Secondary hyperparathyroidism due to chronic kidney disease or prolonged hypocalcemia. 3) Patient refuses to undergo parathyroid surgery. 4) Pregnant or lactating women. 5) Previous history of thyroid or parathyroid surgery. The radioactive drugs used in this study have been approved by an independent ethics committee and reviewed and approved by the institutional review board of Jilin University China-Japan Union Hospital. All patients provided informed consent and voluntarily signed informed consent forms.

### PET image interpretation

^11^C-choline was prepared by the PET Center of the Nuclear Medicine Department at Jilin University China-Japan Union Hospital. The 11C isotope was produced using a medical cyclotron (HM-10, Sumitomo, Japan). The synthesis of the drug involved the transfer of 11C-CO2 to a multifunctional automated synthesis module, radiochemical purity exceeding 95%. All patients received ^11^C-choline via intravenous injection into the cubital vein, with a dosage of 185 MBq. PET/CT imaging was performed 10 minutes after injection (uMI 780, United Imaging Healthcare, China). Patients were positioned in a supine posture during the examination, which covered the neck and chest areas. CT scans were performed with the following parameters: tube voltage of 120 kV, tube current of 40 mA, slice thickness of 5 mm, and a scanning matrix of 512×512. Immediately after the CT scan, PET imaging of the same area was conducted. The duration of each bed position was 3 minutes, with a total of 2 bed positions scanned, and a slice thickness of 5 mm. The acquired PET data were reconstructed using the Ordered Subset Expectation Maximization (OSEM) algorithm. Additionally, attenuation correction of the PET images was performed using CT scan data. All collected images were uniformly transmitted to the uWS-CT software (United Imaging Healthcare, China) for co-registration and fusion.

### Single isotope dual phase 99mTc-sestamibi scan and SPECT/CT scan

After adding 1.5ml of Na99mTcO4 solution to the MIBI freeze-dried reagent vial and thorough mixing, labeling was completed with a 30-minute water bath at 100℃. The radiochemical purity was determined by Thin Layer Chromatography (TLC) which exceeded 95%. Imaging was performed using an SPECT/CT device (Precedence, Amsterdam, Philips Healthcare) equipped with dual-head low-energy high-resolution collimators. All patients received intravenous injections of ^99m^Tc-MIBI via the cubital vein, with a dosage of 740 MBq. Early and delayed planar static imaging was conducted at 15 and 120 minutes post-injection, respectively. Patients were positioned in a supine posture during the examination. They remained still throughout the scanning process, and the detectors were positioned as close as possible to the patient's body. The imaging field covered the neck and chest regions. The image acquisition matrix was set at 256×256. Immediately following the delayed image acquisition, SPECT/CT tomographic imaging was performed. SPECT imaging was conducted with a matrix size of 128×128. Each detector head rotated 180°, with 6°/frame, 20 seconds/frame. Following SPECT imaging, a CT scan of the same region was performed with a tube voltage of 130 kV, tube current of 120 mA, and a slice thickness of 2 mm. All acquired images were reconstructed using the OSEM algorithm with a total of 10 subsets and 2 iterations. After image acquisition, SPECT/CT image reconstruction was performed using the Extended Brilliance Workspace (Astonish SPECT algorithm, Philips Healthcare).

### Neck ultrasound

Neck ultrasound examinations were conducted by the ultrasound department at Jilin University China-Japan Union Hospital using the ultrasound scanner (Resona 8, Mindray, China) equipped with a high-frequency linear array transducer operating at 12 MHz. Patients were instructed to assume a supine position before the examination. The examination encompassed the area from the upper jawbone to the supra-sternal notch and extended bilaterally to the region just before the posterior edge of the sternocleidomastoid muscle.

### Imaging analysis

The ^99m^Tc-MIBI dual-phase planar scintigraphy, ^99m^Tc-MIBI SPECT/CT, and ^11^C-choline PET/CT images were independently reviewed by two senior nuclear medicine physicians in a double-blind manner. In cases of disagreement, a consensus was reached through further review and discussion. The criteria for determination were as follows: 1) Early-phase focal abnormal radioactive uptake was visible in planar imaging, and the lesion remained clearly visible in the delayed phase. 2) Within the scan range, CT images showed nodular soft tissue density, and ^99m^Tc-MIBI SPECT imaging showed increased radioactive abnormalities in the lesion. 3) Within the scan range, CT images showed nodular soft tissue density, and ^11^C-choline PET imaging showed increased radioactive abnormalities in the lesion. In all of these cases, the results were considered positive; otherwise, they were considered negative. The collected ultrasound images were independently evaluated in a double-blind manner by two senior ultrasound department physicians. During the examination, if suspicious parathyroid lesions were identified, the anatomical location, morphology, internal and posterior echo intensity, edges, relationship with surrounding tissues, presence of calcification, cystic changes, and other parameters of the lesions were observed. Furthermore, color Doppler flow imaging was used to assess the blood supply in and around the lesions. The number and diameter of the lesions were also recorded.

All patients included in the study were counted for a total of 4 parathyroid glands on both sides. For each gland, we define the following: True Positive: Surgical removal of the lesion is consistent with the anatomical location of the lesion found in the imaging results, and this is confirmed by postoperative pathological results, with the patient's biochemical levels showing short-term relief after surgery. False Positive: During surgery, the lesion suggested by the imaging examination results was not found, or postoperative pathology confirmed that the lesion was not parathyroid tissue, and the patient's biochemical levels showed short-term relief after surgery. True Negative: The glands were not considered lesions during both the imaging examination and surgical exploration. False Negative: The imaging results were negative, but the lesion at that location was confirmed as a parathyroid lesion by postoperative pathology, or the patient did not experience relief in biochemical levels after surgery.

### Statistical analysis

Statistical analysis was conducted using SPSS 25.0 software, with measurements expressed as mean ± standard deviation (SD). The chi-square test was employed to compare diagnostic sensitivity, specificity, and other parameters across various imaging modalities. Concurrently, we constructed Receiver Operating Characteristic curves (ROCs) and utilized z-tests to compare the diagnostic efficacy of distinct imaging techniques. Patients were classified into positive and negative groups based on the findings from ^99m^Tc-MIBI SPECT/CT imaging. To evaluate distinctions between these two groups, t-tests were employed to analyze preoperative blood calcium, PTH, and lesion length. Statistical significance was determined when the significance level was P < 0.05.

## Results

A total of 32 patients were included in this study. Among them, there were 5 males and 27 females, with an average age of 49.5±14.7 years. The preoperative blood calcium level was 2.8±0.3 mmol/L, and the preoperative PTH level was 270.9±215.1 pg/ml. The lesion size had a mean long diameter of 10.3±5.2 mm. All 32 patients underwent BNE surgery, during which a total of 35 lesions were excised. Among these lesions, 32 were parathyroid adenomas, 2 were parathyroid hyperplasias, and 1 was a thyroid papillary carcinoma. In 30 patients, a single lesion was found, while 2 patients had multiple lesions.

**Figure 1:**
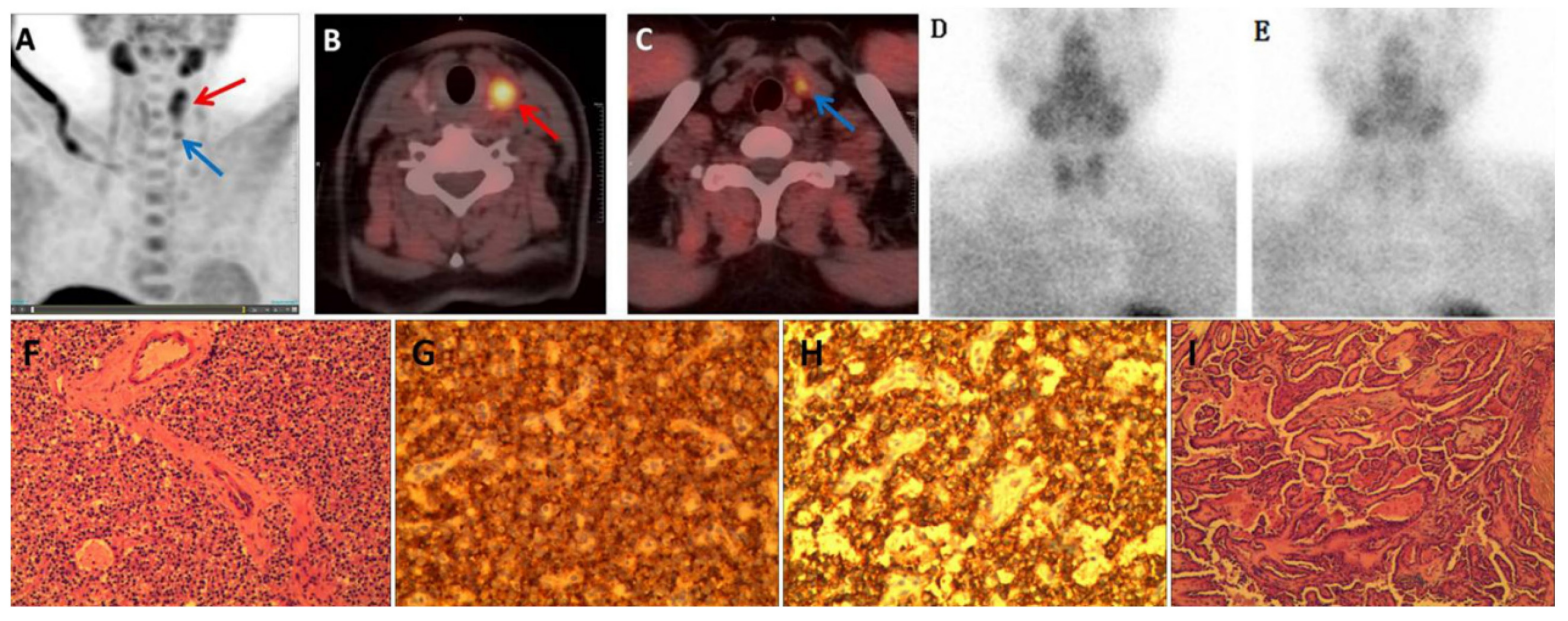
(A) Maximum intensity projection image of ^11^C-choline showing increased radioactive distribution in the left thyroid lobe (red arrow) and below the left thyroid lobe (blue arrow); (B, C) Fusion imaging of ^11^C-choline PET/CT showing increased radioactive uptake in the left thyroid lobe nodule (red arrow) and a nodule below the lower pole of the left thyroid lobe (blue arrow); Dual-phase planar imaging with ^99m^Tc-MIBI showing no increased radioactive foci in the early phase (D) or delayed phase (E); (F) Parathyroid adenoma; (G) Immunohistochemistry for PTH (+); (H) Immunohistochemistry for synaptophysin (syN +); (I) Thyroid papillary carcinoma with infarction.

Among all 23 cases with positive findings in ^99m^Tc-MIBI SPECT/CT imaging, there was notable uptake of ^11^C-choline. Furthermore, ^11^C-choline PET/CT detected 7 lesions that appeared negative in ^99m^Tc-MIBI imaging in patients with PHPT. All these lesions had long diameters less than 10.0 mm. ^11^C-choline PET/CT imaging produced 3 false-positive results. One patient presented with PHPT (long diameter 8.7 mm) and concurrent thyroid papillary carcinoma (long diameter 12.3 mm). Both PHPT and thyroid tumor lesions exhibited notable ^11^C-choline uptake, despite both being negative on ^99m^Tc-MIBI imaging. In the diagnostic process using ^11^C-choline PET/CT, the thyroid papillary carcinoma was erroneously identified as intrathyroidal parathyroid hyperplasia, as illustrated in Figure 1. Two other cases of false-positive results in ^11^C-choline imaging did not found lesions during surgery, and the patient's PTH decreased to normal levels during postoperative follow-up. In four cases, ^11^C-choline PET/CT imaging yielded false-negative results. Pathological examination confirmed that 3 of these cases were parathyroid adenomas, and 1 was parathyroid hyperplasia. Among the parathyroid adenomas, 3 exhibited notable cystic changes and necrosis, while the long diameter of the parathyroid hyperplasia lesion was 2.8 mm.

The sensitivity of ^11^C-choline PET/CT, neck ultrasound, ^99m^Tc-MIBI dual-phase planar imaging, and ^99m^Tc-MIBI SPECT/CT for the diagnosis of parathyroid lesions were 88.2%, 52.9%, 58.8%, and 67.6%, respectively. The specificity was 96.8%, 95.7%, 96.8%, and 95.7%, respectively. The positive predictive values were 90.9%, 81.8%, 86.9%, and 85.2%, respectively. The negative predictive values were 95.7%, 84.9%, 86.7%, and 89.1%, respectively. Notably, the sensitivity of ^11^C-choline PET/CT was higher than that of neck ultrasound, ^99m^Tc-MIBI dual-phase planar imaging, and ^99m^Tc-MIBI SPECT/CT, and this difference was statistically significant (P < 0.05). However, there were no significant statistical differences in specificity, positive predictive value, and negative predictive value, as shown in Table 1.

**Figure 2:**
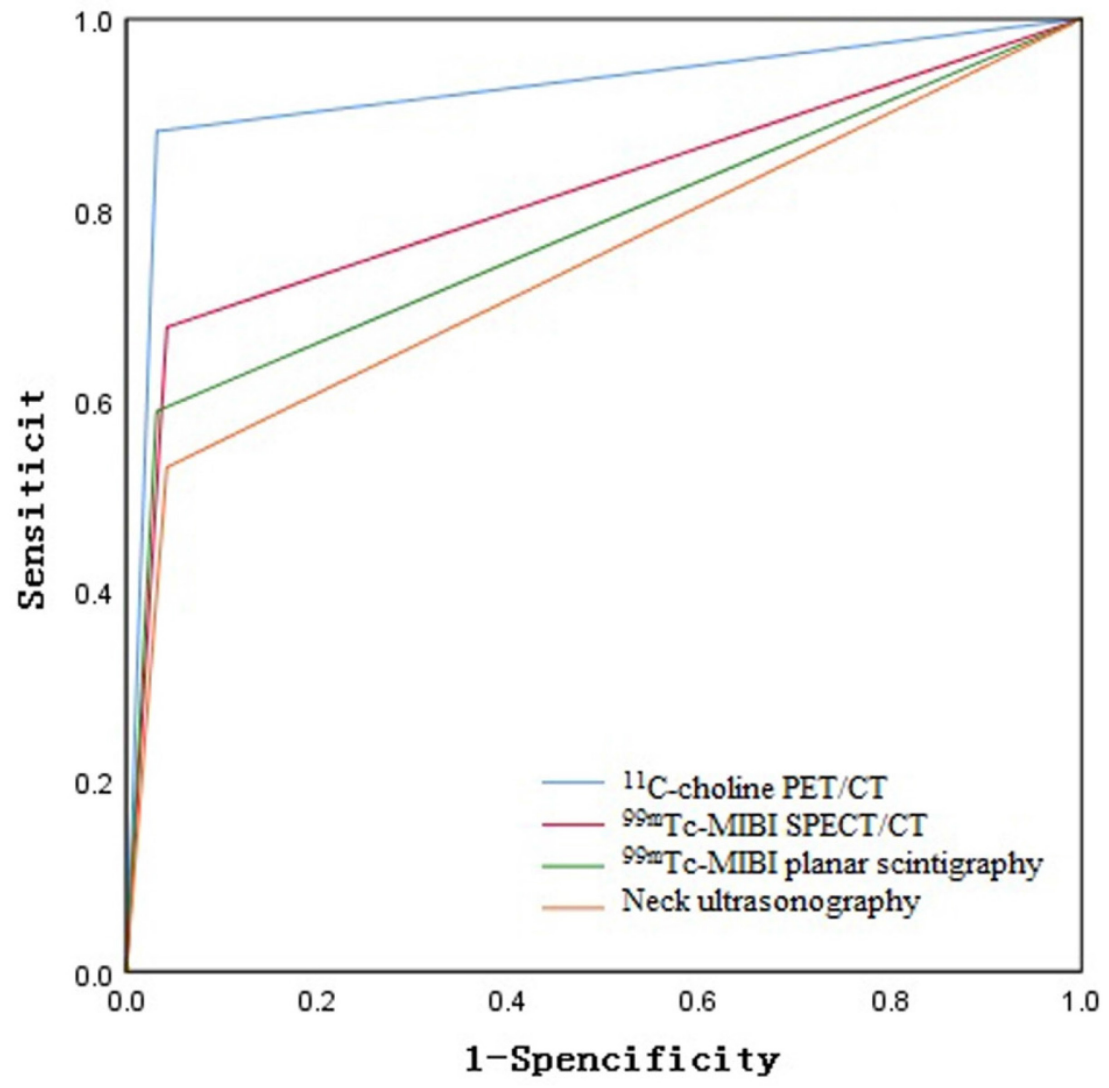
ROC Curve Analysis of the Diagnostic Efficacy of ^11^C-choline PET/CT, Neck Ultrasound, ^99m^Tc-MIBI planar scintigraphy, and ^99m^Tc-MIBI SPECT/CT.

Based on postoperative pathology and follow-up results, the ROC curve areas for ^11^C-choline PET/CT, neck ultrasound, ^99m^Tc-MIBI dual-phase planar imaging, and ^99m^Tc-MIBI SPECT/CT were 0.925, 0.743, 0.778, and, 0.817 respectively, as shown in Figure 2. The diagnostic efficacy of ^11^C-choline PET/CT was superior to that of ^99m^Tc-MIBI SPECT/CT, ^99m^Tc-MIBI dual-phase imaging, and neck ultrasound (Z=3.156, P < 0.05; Z=3.327, P < 0.05; Z=3.955, P < 0.05) (Figure 1). However, there were no statistically significant differences in diagnostic efficacy among ^99m^Tc-MIBI SPECT/CT, ^99m^Tc-MIBI dual-phase imaging, and neck ultrasound (P > 0.05).

According to the results of ^99m^Tc-MIBI SPECT/CT imaging, there were 20 patients in the positive group and 12 patients in the negative group. There was no statistically significant difference in preoperative blood calcium and PTH levels between the two groups (2.87 ± 0.30 vs. 2.63 ± 0.32, 308.36 ± 250.44 vs. 205.21 ± 103.49, P > 0.05). However, patients in the positive group had significantly larger lesion diameters compared to the negative group, and this difference was statistically significant (12.61 ± 4.62 vs. 5.92 ± 1.50, P < 0.05), as shown in Table 2.

## Discussion

Primary hyperparathyroidism is the most common endocrine disorder, following diabetes and hyperthyroidism 1. In this study, a total of 32 cases of parathyroid adenomas and 2 cases of parathyroid hyperplasia were identified. The high incidence of parathyroid adenomas is consistent with previous literature 6. In recent years, minimally invasive parathyroidectomy has gained widespread acceptance among surgeons. In comparison to traditional bilateral neck exploration, its advantages include minimal trauma, shorter surgical duration, lower risk of complications, and faster postoperative recovery. This underscores the importance of precise preoperative localization of the lesion for the success of the surgery. Choline is a major component of cell membranes and also a substrate for the synthesis of phosphatidylcholine. The upregulation of phosphatidylcholine-dependent choline kinase is positively correlated with the excessive secretion of PTH by parathyroid glands. This results in the substantial uptake of ^11^C-choline by the cell membranes of proliferating parathyroid cells, making it applicable for PET/CT imaging 7.

In this study, the sensitivity of ^11^C-choline PET/CT for diagnosing PHPT was 88.2%, with a specificity of 96.8%, a positive predictive value of 90.9%, and a negative predictive value of 95.7%. These results are similar to the findings of Johan Benjamin et al. 8. In another study by Treglia G et al., the sensitivity and positive predictive value of ^11^C-choline PET/CT imaging reached 92% 9. The slightly lower sensitivity in our study compared to the mentioned research could be attributed to the smaller number of patients included in our study. 18F-choline PET/CT can detect more small lesions in PHPT compared to 99mTc MIBI imaging, but there is no significant correlation with the pathological composition of the lesion tissue 10, 11. In our study, ^11^C-choline PET/CT imaging detected an additional 7 lesions with a long diameter of less than 10.0 mm compared to ^99m^Tc-MIBI imaging, indicating its advantage in detecting small lesions. One patient in our study had thyroid papillary carcinoma concomitant with PHPT, and the thyroid cancer lesion showed significant uptake of ^11^C-choline, resulting in a false-positive result. Previous studies have shown that the activation of the choline phosphorylation pathway in thyroid cancer tumor cells, along with the overexpression of choline acetylation pathway-related proteins, may be the main reason for their ability to take up an excessive amount of ^11^C-choline 12, 13. Additionally, enlarged lymph nodes, thymomas, and granulomatous diseases can also lead to false-positive results on ^11^C-choline PET/CT imaging 14-16. In our study, there were 4 false-negative results on ^11^C-choline PET/CT imaging. Three of these had significant cystic changes and necrosis within parathyroid adenomas, lacking blood flow in the cystic and necrotic tissues, which could be the reason for the false-negative results. One case of parathyroid hyperplasia had a long diameter of less than 2.8 mm, and the small size of the lesion may have contributed to the false-negative result. In previous studies, due to the unavailability of pathological results for unresected glands, there is a lack of relevant research on the specificity and negative predictive value of ^11^C-choline PET/CT imaging. In our study, surgeons did not explore all parathyroid glands, and in the statistical process, we calculated based on a total of 4 parathyroid glands per patient, considering the unexplored glands as true negative results. Furthermore, we combined the follow-up information of the patients to infer the number of true negative glands. Therefore, the specificity and negative predictive value in our study should not be interpreted as true results but rather as approximations of the diagnostic efficacy of imaging examinations. The results indicate that the specificity and negative predictive value of ^11^C-choline PET/CT imaging were not significantly different from neck ultrasound, ^99m^Tc-MIBI dual-phase planar imaging, and ^99m^Tc-MIBI SPECT/CT.

Compared to the clinical routine of neck ultrasound imaging, ^99m^Tc-MIBI imaging has a higher diagnostic value for parathyroid lesions 17. In this imaging modality, ^99m^Tc-MIBI is primarily taken up by acidophilic cells rich in mitochondria. Therefore, in addition to the size of the parathyroid lesion, the accuracy of the examination is also determined by factors such as the blood flow, main cell content, and mitochondrial activity within the lesion 18. In our study, there was no significant difference in preoperative blood calcium and PTH levels between patients with positive and negative ^99m^Tc-MIBI uptake. However, patients in the positive group had significantly larger lesion diameters than those in the negative group. This difference may be related to the limited detection rate of smaller lesions by SPECT imaging.

^11^C-choline PET/CT combines functional and anatomical imaging. Despite the short half-life of 11C, making it less suitable for routine examinations, its accurate localization of PHPT lesions can reduce the economic burden on patients from reoperation and readmission to some extent. This potential positions it as a first-line diagnostic approach. However, due to the relatively small sample size in our study and the lack of in-depth analysis of diagnostic efficacy for different pathological types of PHPT lesions, further research with larger, multicenter studies is needed to validate the preoperative diagnostic value of ^11^C-choline PET/CT.

## Conclusion

^11^C-choline PET/CT exhibits superior preoperative diagnostic efficacy in patients with primary hyperparathyroidism compared to neck ultrasound, ^99m^Tc-MIBI dual-phase planar imaging, and ^99m^Tc-MIBI SPECT/CT. Lesion size may be the primary factor influencing the sensitivity of ^99m^Tc-MIBI imaging.

## Figures and Tables

**Table 1:** Diagnostic Efficacy of ^11^C-choline PET/CT and Neck Ultrasound, ^99m^Tc-MIBI planar imaging, and ^99m^Tc-MIBI SPECT/CT

	Sensitivity (%)	Specificity (%)	PPV (%)	NPV (%)
^11^C-choline PET/CT	88.2*	96.8	90.9	95.7
Neck ultrasound	52.9	95.7	81.8	84.9
^99m^Tc-MIBI planar imaging	58.8	96.8	86.9	86.7
^99m^Tc-MIBI SPECT/CT	67.6	95.7	85.2	89.1

* indicates that the sensitivity of ^11^C-choline PET/CT is significantly higher than that of neck ultrasound, dual-phase planar imaging with ^99m^Tc-MIBI, and ^99m^Tc-MIBI SPECT/CT (p < 0.05); PPV: positive predictive values, NPV: negative predictive values.

**Table 2:** Comparison of PTH, calcium, and lesion diameter between the ^99m^Tc-MIBI SPECT/CT positive and negative imaging groups

^99m^Tc-MIBI SPECT/CT	n	PTH (pg/ml)	Calcium (mmol/L)	Lesion diameters (mm)
positive	20	305.36±250.44	2.87±0.30	12.61±4.62
negative	12	205.21±103.49	2.63±0.32	5.92±2.69
P		>0.05	>0.05	<0.05
